# Using deep learning generated CBCT contours for online dose assessment of prostate SABR treatments

**DOI:** 10.1002/acm2.70098

**Published:** 2025-04-23

**Authors:** Conor Sinclair Smith, Isabelle Gagne, Karl Otto, Carter Kolbeck, Joshua Giambattista, Abraham Alexander, Sonja Murchison, Andrew Pritchard, Erika Chin

**Affiliations:** ^1^ Department of Physics and Astronomy University of Victoria Victoria British Columbia Canada; ^2^ Department of Medical Physics BC Cancer Victoria Victoria British Columbia Canada; ^3^ Radformation Inc New York New York USA; ^4^ Allan Blair Cancer Centre Saskatchewan Cancer Agency Regina Saskatchewan Canada; ^5^ Department of Radiation Oncology BC Cancer Victoria Victoria British Columbia Canada; ^6^ Department of Computer Science, Mathematics, Physics, and Statistics University of British Columbia–Okanagan Kelowna British Columbia Canada

**Keywords:** autocontouring, autosegmentation, inter‐fraction variability, organs‐at‐risk, prostate SABR

## Abstract

Prostate Stereotactic Ablative Body Radiotherapy (SABR) is an ultra‐hypofractionated treatment where small setup errors can lead to higher doses to organs at risk (OARs). Although bowel and bladder preparation protocols reduce inter‐fraction variability, inconsistent patient adherence still results in OAR variability. At many centers without online adaptive machines, radiation therapists use decision trees (DTs) to visually assess patient setup, yet their application varies. To evaluate our center's DTs, we employed deep learning‐generated cone‐beam computed tomography (CBCT) contours to estimate daily doses to the rectum and bladder, comparing these with planned dose‐volume metrics to guide future personalized DT development. Two hundred pretreatment CBCT scans from 40 prostate SABR patients (each receiving 40 Gy in five fractions) were auto‐contoured retrospectively, and daily rectum and bladder doses were estimated by overlaying the planned dose on the CBCT using online rigid registration data. Dose‐volume metrics were classified as “no”, “minor”, or “major” violations based on meeting preferred or mandatory goals. Twenty‐seven percent of fractions exhibited at least one major bladder violation (with an additional 34% minor), while 14% of fractions had a major rectum violation (10% minor). Across treatments, five patients had recurring bladder V37 Gy major violations and two had rectum V36 Gy major violations. Bowel and bladder preparation significantly influenced OAR position and volume, leading to unmet mandatory goals. Our retrospective analysis underscores the significant impact of patient preparation on dosimetric outcomes. Our findings highlight that DTs based solely on visual assessment miss dose metric violations due to human error; only 23 of 59 under‐filled bladder fractions were flagged. In addition to the insensitivity of visual assessments, variability in DT application further compromises patient setup evaluation. These analyses confirm that reliance on visual inspection alone can overlook deviations, emphasizing the need for automated tools to ensure adherence to dosimetric constraints in prostate SABR.

## 1 INTRODUCTION

Prostate Stereotactic Ablative Body Radiotherapy (SABR) is an ultra‐hypofractionated radiotherapy (RT) treatment where dose is delivered in 2–7 fractions. This treatment strategy has seen increased usage in recent years with advances in intensity modulation, image guidance, and rectal spacers.^1^ With such high ablative doses per fraction and steep dose gradients, small errors in patient preparation or setup can potentially result in the delivery of higher doses to Organs‐at‐risk (OARs), increasing the probability of toxicity. Two OARs of primary concern in prostate SABR are the adjacent bladder and rectum whose shape and volume are highly susceptible to change. To ensure consistency in OAR volumes from the initial planning CT (pCT) to subsequent treatment fractions, bowel, and bladder preparation protocols are commonly used that are designed to maintain a consistently full bladder and empty rectum. Despite this strict protocol, it is impossible to completely eliminate all OAR volume variations due to variability in patient compliance, which can lead to higher OAR doses than planned.^2^

**FIGURE 1:**
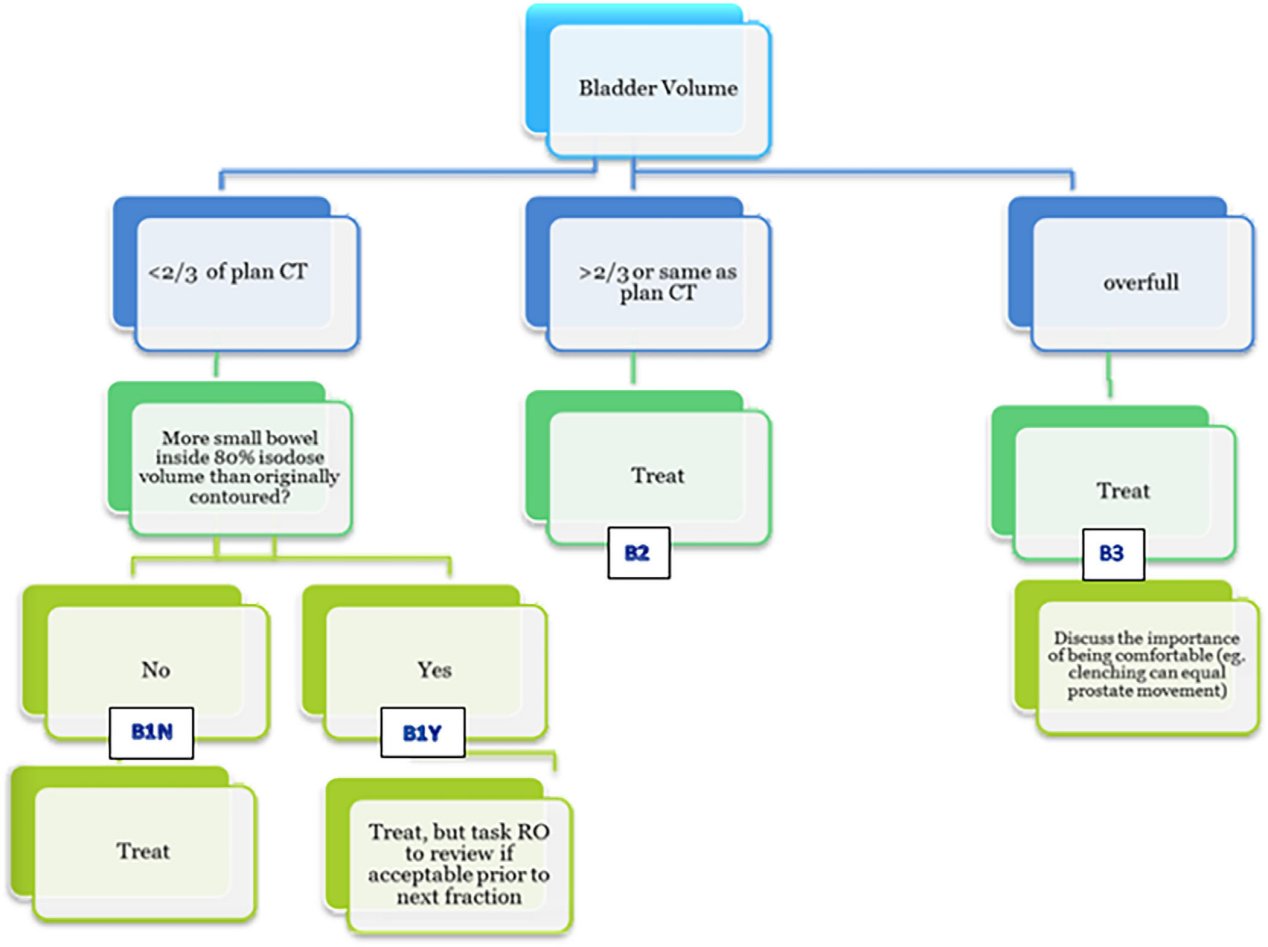
Bladder decision tree for prostate SABR patients. SABR, stereotactic ablative body radiotherapy.

**FIGURE 2:**
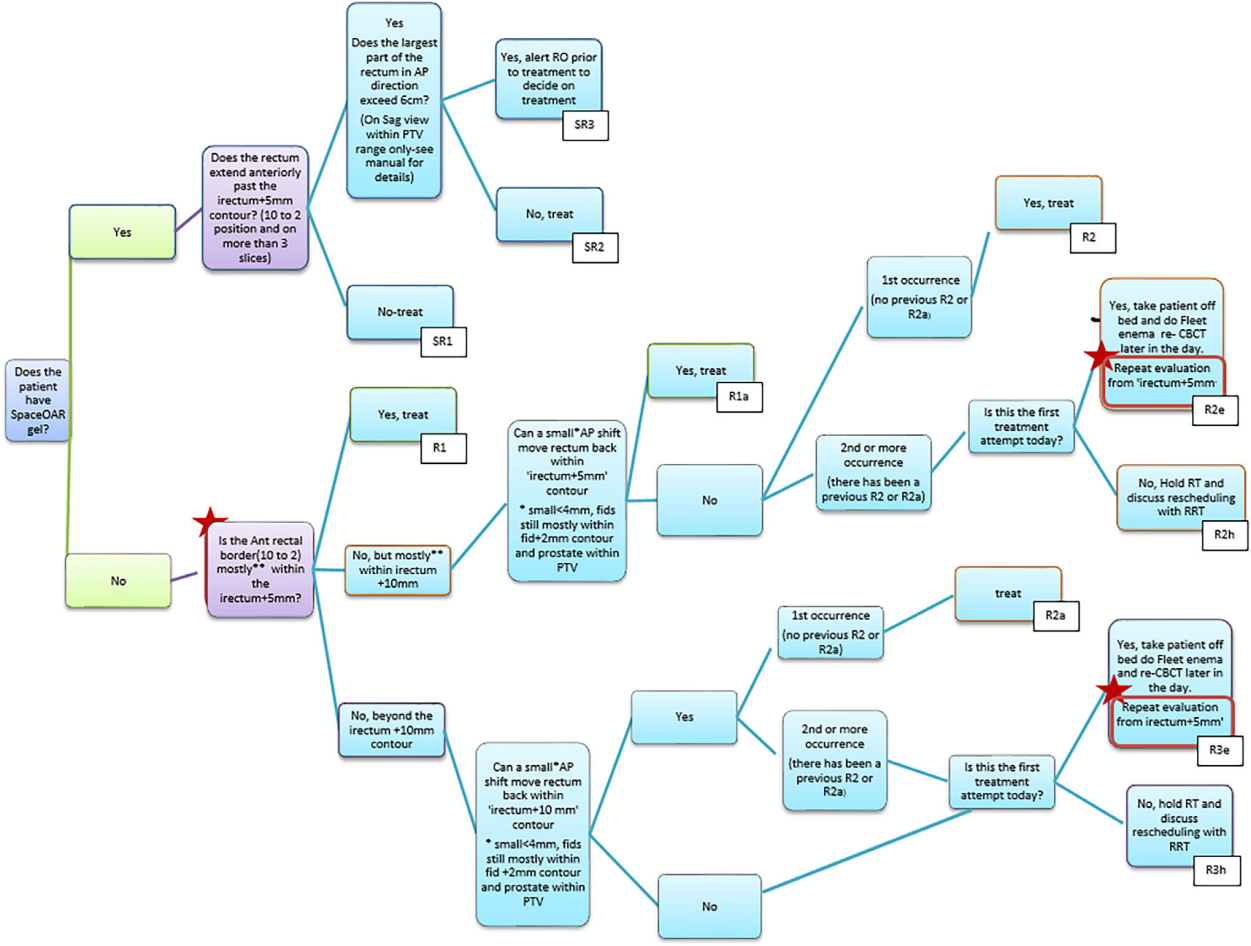
Rectum decision tree for prostate SABR patients. SABR, stereotactic ablative body radiotherapy.

To account for this reduced but remaining daily anatomical variation, BC Cancer Victoria's radiation therapists use specially designed bladder (Figure 1) and bowel (Figure 2) decision trees (DTs) to visually assess patient pre‐treatment Cone‐Beam Computed Tomography (CBCT) images and determine if the patient preparation and setup is acceptable. Originally developed for the ASSERT clinical trial,^3^ the primary goal of these DTs was to ensure patient safety while enhancing treatment efficiency by avoiding unnecessary patient removal from the treatment table. These DTs were developed based on dose‐volume histogram (DVH) metrics from simulated OAR bladder and rectum distensions and rotations on a selected population of previously treated SABR prostate patients.^4^ Following the trial, further modifications were made to permit therapists to make decisions, especially as patient volume grew, thereby relieving physicists and ROs from being present at every treatment session. Various thresholds for volume and distension of the rectum and bladder were set in the DTs to trigger different actions for therapists such as adjusting the couch in the anterior/posterior direction, tasking the radiation oncologist (RO) for review, or even canceling and rescheduling the patient. These thresholds acted as dosimetric surrogates to minimize the probability that the delivered treatment would violate OAR dose constraints.

Since implementation in our clinic, the DTs have allowed therapists to drive SABR prostate treatments and freed clinical resources but there are several disadvantages to their use. The DTs used in prostate SABR patient setup are qualitative in nature, complicated, and time‐consuming to apply at the machine, furthermore, they are not personalized to the patient's specific anatomy. This work aims to assess the current efficacy of our clinic's DTs by developing a simplified automated tool for online patient pre‐treatment dose assessment. While prostate radiotherapy studies looking at planned versus delivered doses have been done,^5^, ^6^ this study is unique in that the dose difference is being evaluated in the context of whether the application of our clinic's DTs for prostate patient setup helped avoid violation of OAR dose constraints. Clinical DTs have been implemented across the medical sciences to aid clinicians in making consistent decisions.^7^, ^8^, ^9^ To the authors’ knowledge, no publications exist specifically examining the use of DTs in radiation therapy patient setup. The eventual goal is to use the tools developed in this study to refine the clinic's current qualitative DT for patient setup into a quantitative DT that can provide rapid insights into expected OAR dose based on patient daily patient setup.

## 2 METHODS

### 2.1 Patient preparation, CT simulation, and treatment planning & delivery

The pCT scans, CBCT images, and treatment plans utilized for the study were from 40 patients treated for prostate cancer using a SABR technique, delivering 40 Gy in five fractions to the prostate gland. Each patient had SpaceOAR gel placed between the prostate and rectum, and three fiducial markers implanted in the prostate, as is standard for prostate SABR at our institution. Prior to both the pCT and each treatment fraction, patients were instructed to follow an empty bowel and full bladder preparation protocol to reduce the geometric inter‐fraction variability of the rectum and bladder. pCT scans were performed using a GE Optima CT580 unit (GE Healthcare, Chicago, Illinois, USA) and acquired using a 0° helical scan method, 0.25 cm slice thickness, 120 kVp, 700 mA tube current, and 12.39 s exposure time.

All treatments were planned in Eclipse 15.6 (Varian—A Siemens Healthineers Company, Palo Alto, USA) and delivered using a two‐arc VMAT technique and a 0.25 × 0.25 × 0.25 cm dose calculation voxel size. Table 1 outlines the treatment planning metrics that were used for planning 40 Gy/5 fx prostate SABR patients. Treatments were planned using OARs such as the rectum, sigmoid, and bladder have more than one primary goal to be incorporated into treatment planning. Several structures have a secondary goal if the primary goal cannot be met during optimization. Bladder and rectum DVH curves from CBCT contours were evaluated based on these same metrics to categorize them into optimal, minor violation, or major violation.

**TABLE 1:** BC cancer victoria prostate SABR planning goals for 40 Gy/5 fraction prescription.

Structure	Primary goal	Secondary goal
CTV	V40 Gy ≥ 95%	V39.2 Gy ≥ 95%
PTV	V36.25 Gy ≥ 95%	–
Rectum + 5 mm	Dmax ≤ 105%	Dmax ≤ 107%
Rectum	V36 Gy ≤ 1 cm^3^	V36 Gy ≤ 2 cm^3^
	V33 Gy ≤ 10%	V33 Gy ≤ 12.5%
	V29 Gy ≤ 20%	V29 Gy ≤ 22.5%
	V18 Gy ≤ 40%	V18 Gy ≤ 50%
Sigmoid	V30 Gy ≤ 1 cm^3^	–
	V18 Gy ≤ 5 cm^3^	–
Bladder	V37 Gy ≤ 5 cm^3^	V37 Gy ≤ 10 cm^3^
	V36 Gy ≤ 5%	V36 Gy ≤ 10%
	V33 Gy ≤ 10%	V33 Gy ≤ 20%
	V18 Gy ≤ 30%	V18 Gy ≤ 45%
Penile Bulb	V30 Gy ≤ 50%	–
Femoral Heads	V14 Gy ≤ 5%	–

Abbreviation: SABR, stereotactic ablative body radiotherapy.

Treatments were delivered using a Varian TrueBeam medical linear accelerator equipped with Millennium series Multi‐Leaf Collimators and 3‐degrees‐of‐freedom (3‐DOF) treatment couches. Patients were initially aligned using fiducial markers and two orthogonal kV digitally reconstructed radiographs (DRRs). A confirmatory CBCT scan again assessed marker positioning in addition to OAR position and volume, employing dedicated bladder and rectum DTs (Figures 1 and 2). If the anatomy on the CBCT was deemed acceptable, the first arc was delivered. A second set of kV images was taken to assess intra‐fraction motion and corrected for, an inter‐arc CBCT may be taken if the correction was large (>4 mm). Once any potential intra‐fraction motion was corrected, the second treatment arc was delivered. A post‐treatment CBCT was then taken for offline review by a RO.

### 2.2 CBCT OAR auto‐contouring

A prerequisite for obtaining a fast online estimate of the delivered dose is the automated generation of OAR contours on the CBCT. Historically, ATLAS‐based methods have been used for auto‐contouring. However, ATLAS methods score lower Dice Similarity Coefficients (DSC) and higher Hausdorff Distances (HD)^10^, ^11^ and are less geometrically similar to manual contours compared to newer deep‐learning (DL) based methods.^12^, ^13^

Limbus AI software, a commercially available DL based auto‐contouring solution, was selected for use as their AI segmentation models have been deployed and validated elsewhere for a myriad of treatment sites.^14^, ^15^, ^16^, ^17^, ^18^, ^19^, ^20^ A prostate CBCT research version of Limbus (1.5.0‐D2) with models only for rectum and bladder was made available for this study. The Limbus AI models for prostate CBCT were developed using an adapted U‐Net architecture. They were trained on a dataset comprising approximately 500 CBCT scans and 1000 CT scans, each from a different patient, and included both narrow and full‐field views from Varian and Elekta machines.

For this study, contours for rectum and bladder from 200 pre‐treatment prostate CBCT scans were auto‐segmented with the Limbus AI research‐only software. When pre‐treatment CBCT registration data were unavailable, post‐treatment CBCTs were utilized, provided that differences in pre‐treatment and post‐treatment CBCT contours were less than 1 mm at any point along the contour. As the primary focus of this study was on assessing the efficacy of our clinic's DTs which are based on the volumes and locations of the rectum and bladder, the limited number of structures in the initial Limbus beta release was not an issue. Evaluation of PTV and CTV DVH curves was not considered critical to this study as the use of implanted fiducial markers has been shown to provide adequate prostate coverage.^21^, ^22^, ^23^ The Limbus prostate CBCT beta version used in this study has been validated by Radici et al.^24^ with auto‐contours shown to be within interobserver variation. However, a RO reviewed all CBCT auto‐contours in this study.

### 2.3 Calculation of CBCT OAR dose

**FIGURE 3:**
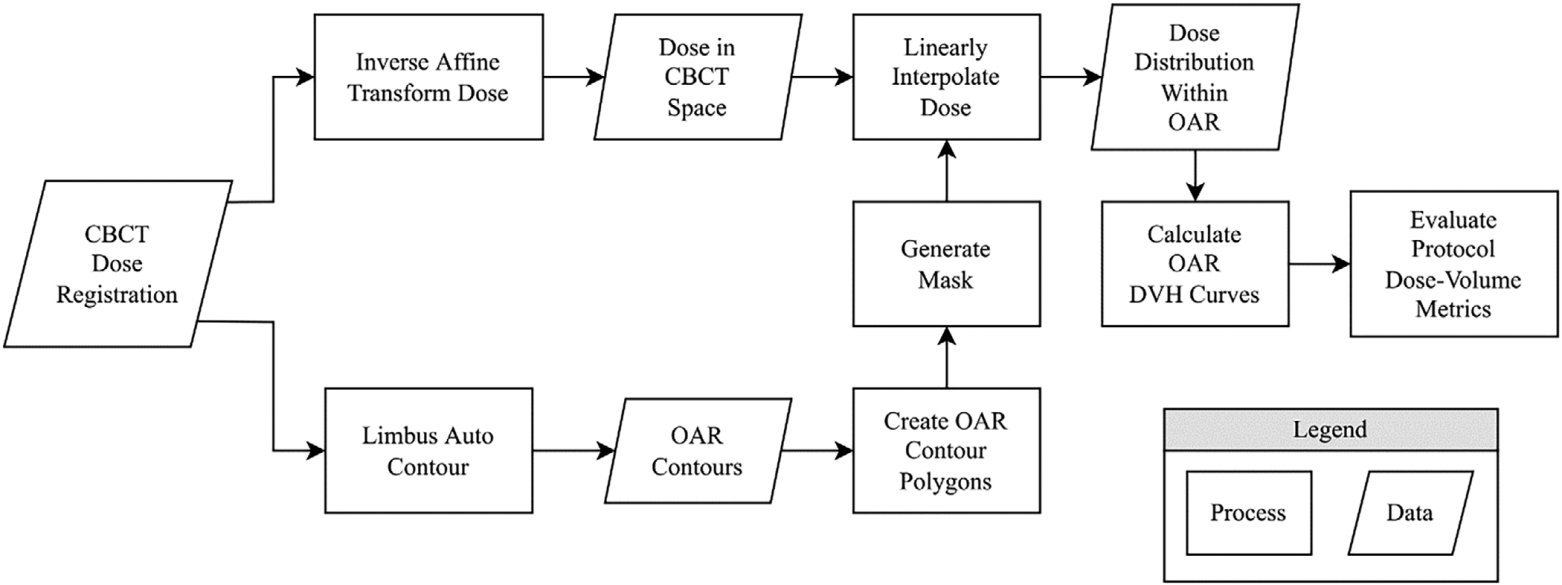
CBCT OAR dose calculation workflow. The position of dose voxels in CBCT‐space is determined using an inverse affine transformation. Meanwhile, Limbus contour data is used to create polygon objects which can be turned into Boolean masks. The dose distribution is linearly interpolated to the position of the CBCT slices, computation time is accelerated by considering only the voxels that are within the OAR. DVH curves can then be calculated and evaluated against the clinical protocol. CBCT, cone‐beam computed tomography; DVH, dose‐volume histogram; OAR, organs at risk.

CBCT rectum and bladder DVH curves were calculated by superimposing the planned dose distribution onto the Limbus‐generated CBCT contours using the online rigid registration data that calculated the 3DOF couch shifts (Figure 3). Dose superimposition was chosen primarily for both efficiency and feasibility reasons. A full dose re‐calculation on the CBCT would be time‐consuming and computationally intensive for an in‐house online patient setup tool. Furthermore, the prostate SABR procedure used involves CBCTs in Spotlight mode, which are insufficient for accurate dose recalculation as the CBCT HU values do not match pCT HU values, and the smaller field of view does not capture the entire patient body contour needed for dose calculation.^25^ We hypothesized that superimposing the dose for prostate SABR would be sufficiently accurate, as the tissues in the immediate vicinity of the prostate are fairly homogeneous, unlike the thorax, where air causes dramatic density changes. Additionally, the VMAT technique spreads the radiation delivery over multiple 360° arcs such that dose inaccuracy from any one angle is averaged out. Overlaying planning structures and planning dose onto the daily patient setup images is a commonly accepted clinical practice as long as the uncertainties of this method are well understood. This technique has been employed in multiple dosimetric studies for prostate, head & neck, and pancreatic patients.^2^, ^26^, ^27^, ^28^, ^29^, ^30^, ^31^ More specifically for prostate cancer, Sharma et al. investigated the validity of using the assumption of shift and deformation‐invariance for dose evaluations by simulating 27 different combinations of patients shifts up to ± 10 mm in the left/right, anterior‐posterior, and superior/inferior and compared it to full dose recalculations on fan‐beam CTs collected over the course of treatment with total dose mapped back to the pCT via deformable image registration (DIR).^32^ They found that the dose cloud assumption of shift and deformation‐invariance introduced less than a 2% error in their evaluated dose metrics.

Once planning dose was overlaid onto daily CBCTs, the evaluation of dose to bladder and rectum was on a per fraction basis. This is a conservative worst‐case approach that is commonly used in clinical practice for online treatment decisions. It assumes the maximum doses to OARs is delivered to the same volume for the entire course of treatment. Applying DIR to accumulate the delivered dose onto the pCT was not used in this study due to its known limitations, including inaccuracies in deformation vector fields (DVFs) caused by soft tissue variability and volume changes.^33^, ^34^ These errors can propagate into dose accumulation, potentially leading to misleading DVH metrics. Furthermore, DIR requires rigorous quality assurance and can be computationally intensive, which does not align with the need to make real‐time treatment decisions at the treatment machine.^25^, ^35^ It is for these reasons that the use of deformable dose accumulation software to inform clinical decisions at the treatment unit is still debated and not widely used by clinicians even on commercial systems with online adaptive capabilities.^35^

### 2.4 Dose shift study

Our clinic's current DT to assess patient rectum before treatment (Figure 2) allows therapists to shift the patient isocenter up to a maximum of 4 mm in the anterior/posterior direction to prioritize rectal sparing. While the Sharma et al. study showed that the dose overlay method was accurate within 2% for relevant dose metrics,^32^ this work further investigated the worst‐case scenario of the rectum with no gas in the pCT then being entirely filled with air at treatment because the SABR dose gradients in this region are especially steep. The examined scenarios are outlined in Table 2. The Reference Distributions column served as the “ground truth,” where dose was recalculated as a final step. The Evaluated Distributions column simulated planned dose superimposition onto treatment contours with a corresponding shift. Due to challenges associated with CBCT dose calculations discussed in Section 2.3, these scenarios were executed on patient CT images that had both target and OARs contoured.

**TABLE 2:** Average gamma passing rate (2%/2 mm criteria) for five dose approximation scenarios. One standard deviation is reported for uncertainty.

	Reference distribution	Evaluated distribution	Average gamma passing rate (10% threshold)	Average gamma passing rate (50% threshold)
Case A	1. Isocenter shifted 4 mm anteriorly 2. Recalculate dose	1. Original planned dose 2. Shift dose 4 mm anteriorly	96.87% ± 0.79%	100.00% ± 0%
Case P	1. Isocenter shifted 4 mm posteriorly 2. Recalculate dose	1. Original planned dose 2. Shift dose 4 mm posteriorly	96.84% ± 0.80%	100.00% ± 0%
Case RA	1. Set rectum contour to air 2. Recalculate dose	1. Original planned dose	99.30% ± 0.52%	99.47% ± 1.21%
Case RA‐A	1. Isocenter shifted 4 mm anteriorly 2. Rectum contour set to air 3. Recalculate dose	1. Original planned dose 2. Shift dose 4 mm anteriorly	96.25% ± 1.49%	99.76% ± 0.76%
Case RA‐P	1. Isocenter shifted 4 mm posteriorly 2. Rectum contour set to air 3. Recalculate dose	1. Original planned dose 2. Shift dose 4 mm posteriorly	96.17% ± 1.35%	98.53% ± 2.01%

Cases A and P simulate shifting the patient 4 mm away from the planned treatment isocenter. An anterior isocenter shift (Case A) is generally applied when the visual assessment of the CBCT indicates that the rectum has moved closer to the target volume, risking higher dose exposure. For study completeness, a 4 mm posterior isocenter shift (Case P) was also evaluated. In cases, A and P, the rectum's electron density is not altered as the results will serve as a baseline to separate out the effect of the 4 mm shift from the rectum filled with air.

Cases RA, RA‐A, and RA‐P included the complicating factor of air in the rectum (RA). Despite the strict bowel preparation protocol, the presence of gas bubbles is not always avoidable. These cases simulated the worst‐case scenario of the entire rectum filled with air at the prostate level, with Case RA‐A and RA‐P involving anterior and posterior isocenter shifts, respectively.

Ten patient CT scans were used to test each of the five scenarios. Dose recalculations were performed using Varian Eclipse AAA v15.6, and average local gamma passing rates were determined using the open‐source PyMedPhys library.^36^ Gamma calculation parameters consisted of a 2% dose difference tolerance, a 2 mm dose distance‐to‐agreement threshold, a 0.125 × 0.125 × 0.125 cm voxel size, and an interpolation fraction of 10, which is the fraction that the distance threshold is divided into for interpolation. This gamma analysis was completed for two‐dose thresholds: one with a threshold of 10% of the maximum dose on the reference distribution to include the majority of patient voxels, as well as 50% to examine only voxels closer to the target volume.

While gamma passing rates offer valuable insights into global dose differences, they are not sensitive to local dose variations near the target that could have clinical implications. Furthermore, gamma analysis metrics typically would not be used to assess patient setup at the treatment console; it is more clinically relevant to determine whether or not target volumes or OARs are meeting certain dose‐volume metrics. For these reasons, the dose‐volume metrics that were used as planning goals (Table 1) were also compared for the Evaluated Distribution and the Reference Distribution. Both average percent difference and average absolute difference were computed for each planning metric.

## 3 RESULTS

### 3.1 Dose shift study—Gamma analysis

The average gamma passing rates for Cases A, P, RA, RA‐A, and RA‐P, as outlined in Table 2, were 96.87%, 96.84%, 99.30%, 96.25%, and 96.17%, respectively. These rates were high, considering the strict 2%/2 mm local gamma criteria. Cases RA‐A and RA‐P had the lowest average gamma passing rates due to both a 4 mm shift and the presence of air in the rectum. The American Association of Physicists in Medicine (AAPM) Task Group 218^37^ recommends gamma passing rates greater than 90% using 3%/2 mm criteria for universal action limits in IMRT QA, and 3%/2 mm ≥ 95% for universal tolerance limits. In all cases examined, the average gamma passing rates were greater than 95% despite the use of a tighter 2%/2 mm criteria. Voxels with gamma values greater than 1 tended to be at the skin in cases that involved shifts, or in the rectum in cases where it was calculated with air.

When repeated using a 50% dose threshold, a notable increase in passing rates was observed. This increase resulted from the exclusion of skin‐level voxels, which typically have larger gamma values. Consequently, the average gamma passing rates rose to 100.00%, 100.00%, 99.47%, 99.76%, and 98.53% for Cases A, P, RA, RA‐A, and RA‐P, respectively.

### 3.2 Dose shift study—Dose volume metrics

When comparing cases using planning dose‐volume metrics (Table 3), several instances of large average percent differences accompanied by small absolute differences were observed, primarily in OAR metrics with small numerical values. Conversely, large absolute differences were sometimes paired with small percent differences in metrics with large numerical values, such as target volumes. No metrics exhibited both large average percent and absolute differences.

**TABLE 3:** Dose distributions compared using planning goals for five dose approximation scenarios.

ROI	Metric	Case A		Case P		Case RA		Case RA‐A		Case RA‐P	
		Avg. % Diff.	Avg. Diff.	Avg. % Diff.	Avg. Diff.	Avg. % Diff.	Avg. Diff.	Avg. % Diff.	Avg. Diff.	Avg. % Diff.	Avg. Diff.
Rectum	V36 Gy (cm^3^)	0.00 ± 0.00	0.00 ± 0.00	1.38 ± 2.25	0.01 ± 0.02	65.92 ± 82.02	0.29 ± 0.69	20.0 ± 60.0	0.00 ± 0.01	30.79 ± 33.96	0.84 ± 1.94
Rectum	V33 Gy (% Vol.)	0.75 ± 1.82	0.00 ± 0.01	0.76 ± 1.18	0.02 ± 0.03	27.99 ± 36.89	0.08 ± 0.07	22.04 ± 31.28	0.10 ± 0.25	7.44 ± 8.61	0.19 ± 1.94
Rectum	V29 Gy (% Vol.)	6.82 ± 19.95	0.00 ± 0.00	0.51 ± 10.64	0.03 ± 0.04	9.51 ± 10.52	0.13 ± 0.17	23.4 ± 59.23	0.03 ± 0.05	4.34 ± 4.41	0.23 ± 0.27
Rectum	V18 Gy (% Vol.)	0.89 ± 1.21	0.04 ± 0.06	0.23 ± 0.32	0.06 ± 0.07	6.61 ± 3.29	0.98 ± 0.81	4.41 ± 3.97	0.41 ± 0.67	6.59 ± 3.19	1.60 ± 0.99
Bladder	V37 Gy (cm^3^)	8.72 ± 12.51	0.45 ± 0.40	9.11 ± 11.66	0.20 ± 0.13	13.77 ± 29.2	0.52 ± 0.89	18.21 ± 31.92	1.03 ± 1.27	12.82 ± 23.15	0.29 ± 0.51
Bladder	V36 Gy (% Vol.)	2.17 ± 0.74	0.12 ± 0.08	3.39 ± 1.55	0.07 ± 0.04	3.28 ± 3.76	0.10 ± 0.11	4.62 ± 3.42	0.22 ± 0.15	3.82 ± 3.86	0.07 ± 0.07
Bladder	V33 Gy (% Vol.)	1.17 ± 0.41	0.09 ± 0.07	1.63 ± 0.70	0.06 ± 0.03	1.40 ± 1.65	0.06 ± 0.07	2.40 ± 1.38	0.17 ± 0.10	1.82 ± 1.67	0.05 ± 0.04
Bladder	V18 Gy (% Vol.)	0.33 ± 0.13	0.08 ± 0.07	0.51 ± 0.23	0.09 ± 0.07	0.64 ± 0.65	0.10 ± 0.08	0.83 ± 0.50	0.17 ± 0.11	0.70 ± 0.64	0.09 ± 0.06
Femur Head L	V14 Gy (% Vol.)	7.77 ± 8.39	0.04 ± 0.04	4.59 ± 4.33	0.04 ± 0.05	2.24 ± 5.40	0.01 ± 0.01	6.43 ± 7.24	0.04 ± 0.04	3.93 ± 3.82	0.03 ± 0.04
Femur Head R	V14 Gy (% Vol.)	4.85 ± 7.03	0.04 ± 0.05	4.13 ± 5.90	0.04 ± 0.05	9.24 ± 17.21	0.04 ± 0.04	12.09 ± 225.5	0.07 ± 0.06	7.65 ± 12.65	0.02 ± 0.02
PTV	D0.003cc (% dose)	6.58 ± 0.62	6.75 ± 0.64	6.02 ± 0.58	6.16 ± 0.61	0.50 ± 0.45	0.53 ± 0.48	7.03 ± 0.74	7.23 ± 0.78	6.37 ± 0.83	6.53 ± 0.87
PTV	D95% (cGy)	0.08 ± 0.08	2.86 ± 2.56	0.47 ± 0.13	16.51 ± 4.36	0.46 ± 0.87	16.79 ± 31.23	0.41 ± 0.74	13.94 ± 25.59	0.46 ± 0.44	16.03 ± 15.42
CTV	D99% (cGy)	0.14 ± 0.08	5.34 ± 3.14	2.35 ± 5.84	84.24 ± 206.43	0.74 ± 1.62	26.81 ± 56.76	0.61 ± 0.87	22.36 ± 29.44	2.93 ± 6.10	104.6 ± 216.1

Considering the rectum V36 Gy metric, Case RA‐P showed the worst average absolute difference at 0.84 ± 1.94 cm^3^ and the largest singular difference at 6.60 cm^3^. This case, however, is considered clinically unrealistic as the dose would never be shifted toward the rectum. The worst of the “realistic” scenarios was Case RA, with an absolute average difference of 0.29 ± 0.69 cm^3^, which was not considered clinically significant by our ROs. For the rectum V18, V29, and V33 Gy relative metrics, the absolute differences were mostly less than 1%, with a maximum of 1.6% for Case RA‐P's rectum V18 Gy, which was considered acceptable and within tolerance limits.

For the bladder, V37 Gy metric, Case RA‐A had the largest average absolute difference at 1.03 ± 1.27 cm^3^, while Case RA‐P recorded the largest singular difference at 3.06 cm^3^. Neither was considered clinically significant by our ROs. The remaining cases had differences of less than 0.5 cm^3^ for the bladder V37 Gy metric. The V18, V33, and V36 Gy bladder metrics all had an average absolute difference of 0.22% or less.

Both left and right femur heads showed absolute differences below 0.07%. For target volumes, the PTV D95% metric's average percent difference was under 0.5%, within acceptable limits. Excluding Case RA, the average percent difference for the D0.003cc metric was 6%–7% for all cases. A volume of 0.003 cm^3^ is approximately 1.5 times larger than the dose calculation resolution of 0.125 × 0.125 × 0.125 cm and requires interpolation to compute. For this reason, the D0.003cc metric is particularly sensitive, small differences in the reference and evaluated distributions may yield large differences in D0.003cc values particularly in steep dose gradient regions. The most significant clinical concern was whether or not any plan's D0.003cc value fell below 90% or exceeded 110%, which they did not. Regarding CTV D99%, Cases P and RA‐P had unacceptable differences of 2.35% and 2.93% however they are not considered clinically realistic scenarios. Other cases remained under 0.75%, aligning with RO criteria. These worst‐case scenario results build on the work by Sharma et al.^32^ and demonstrate that while dose superimposition has inherent limitations compared to full recalculation, it is sufficiently accurate for its intended purpose: as an online aid for therapists to rapidly assess patient setup and flag potential issues before treatment.

### 3.3 DVH curves calculated using CBCT contours

**FIGURE 4:**
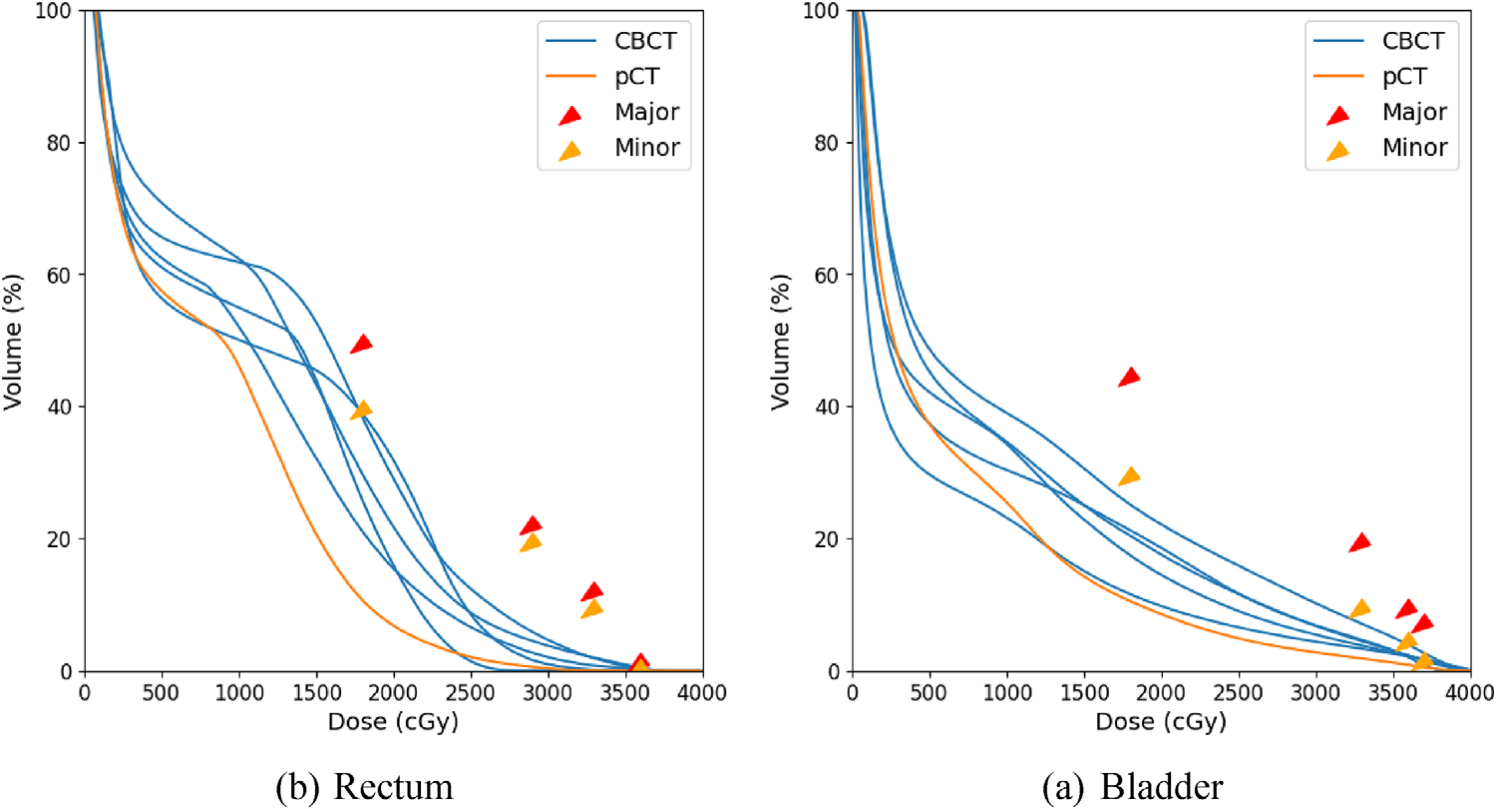
Rectum DVHs from treatment planning, and as calculated using AI‐generated CBCT contours for rectum (a) and bladder (b) from a randomly selected patient. Arrowheads indicate the dose‐volume thresholds used as planning goals. CBCT, cone‐beam computed tomography; DVH, dose‐volume histogram.

Following the methodology in Figure 3, DVH curves for the rectum and bladder were calculated for 40 patients across all their treatment fractions. Bladder contours generated by the research CBCT Limbus auto‐contouring software rarely required any corrections. In the few instances edits were made, they tended to be around the prostate median lobe or bladder diverticulum. Rectum contouring edits were more frequent with approximately 80% of the CBCTs requiring adjustments at the mid‐prostate level. This was likely due to the absence of SpaceOAR gel data in the Limbus training set. The advent of SpaceOAR Vue^38^ promises increased contrast on CT‐based imaging devices and will likely result in improved rectum contours once Health Canada is approved. Once the RO approved all the contours, DVHs were evaluated against planning goals from Table 1, and each metric was classified as being optimal or having minor or major violations. An example is depicted in Figure 4, which shows rectum and bladder DVHs from the pCT and all five CBCTs for a randomly selected patient.

### 3.4 Bladder

**FIGURE 5:**
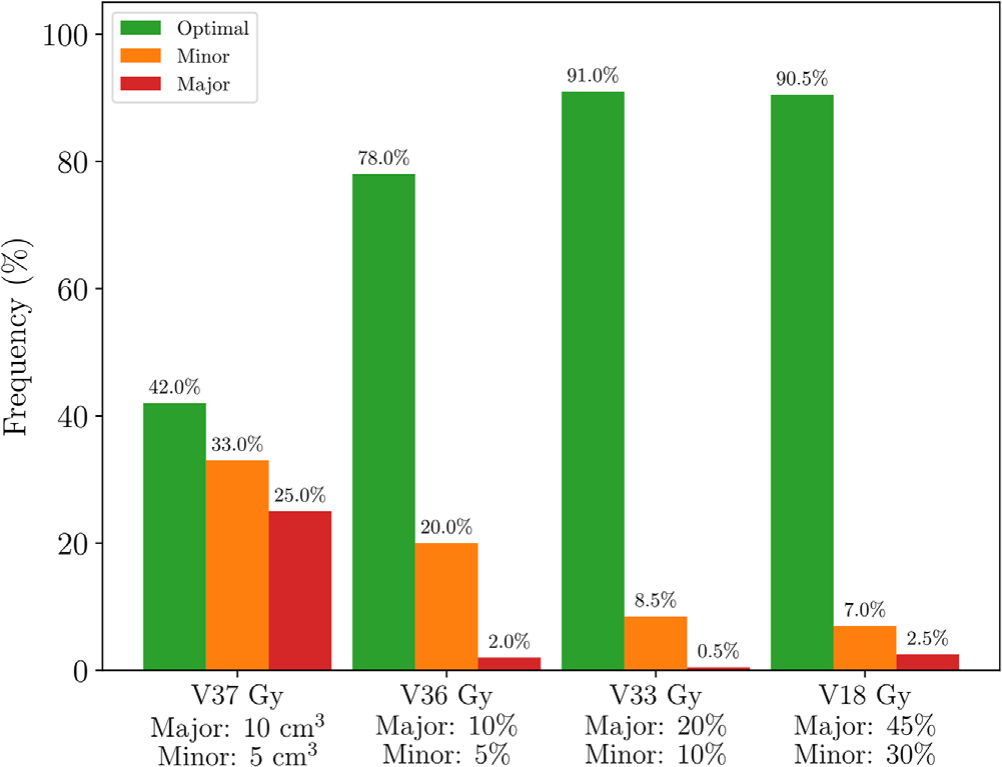
Classification of dose‐volume metrics using daily CBCT bladder contours. CBCT, cone‐beam computed tomography.

Figure 5 categorizes the 200 analyzed fractions based on bladder dose‐volume constraints. Fractions with any planning goal violations were marked as minor or major. Major violations were below 2.5% for V18, V33, and V36 Gy metrics but escalated to 25% for the V37 Gy metric. Minor violations were also more frequent for V37 Gy, with rates of 33.0%, in contrast to 7.0%, 8.5%, and 20.0% for V18, V33, and V36 Gy. Out of the 200 fractions, 27% had a major violation in at least one bladder metric, whereas 39% of fractions were classified as optimal for all four bladder metrics.

**FIGURE 6:**
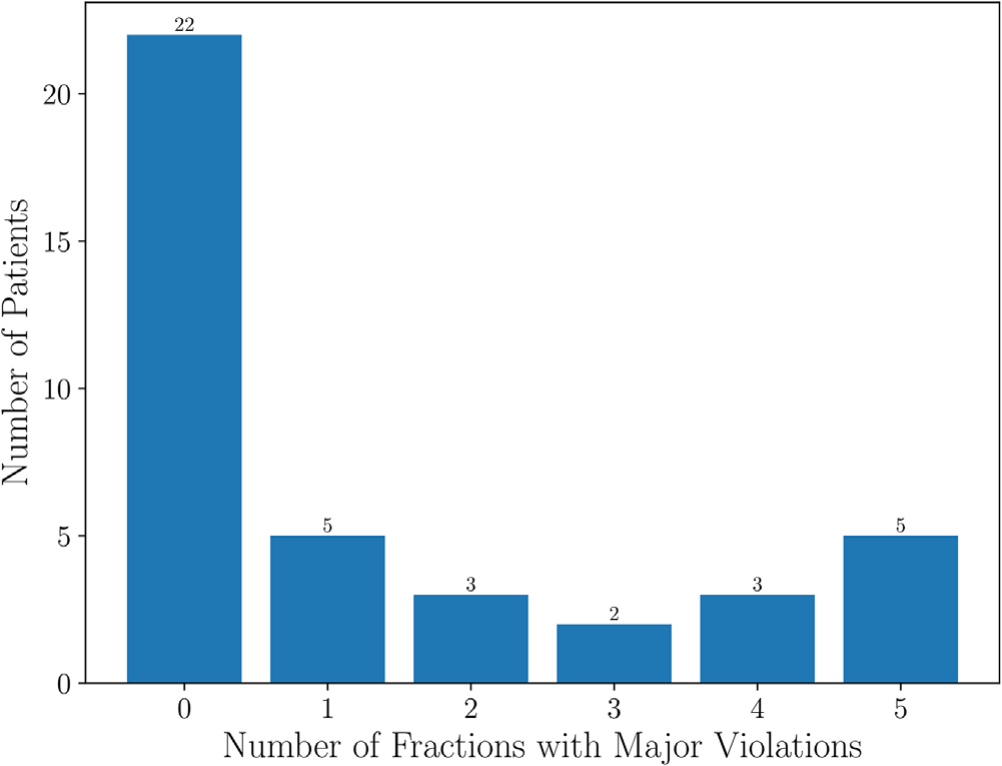
Bladder—Patients by number of major dose violations over the course of their treatment.

At treatment planning, some minor violations were accepted; however no patients had major violations of bladder metrics. The minor violations occurred primarily in the V37 Gy metric where 8/40 patients had a planned minor violation. A single patient had a planned minor violation in the V36 Gy and V33 Gy metrics and two patients with V18 Gy minor violations. Figure 6 shows how many patients received a certain number of major violations over the course of their five‐fraction treatment. Out of 40 patients, only 22 had no bladder major violations. Of concern, five patients experienced major violations in every fraction; four of them consistently in the V37 Gy metric, which correlates with worse Expanded Prostate Cancer Index Composite (EPIC) quality of life (QOL) score.^39^

**FIGURE 7:**
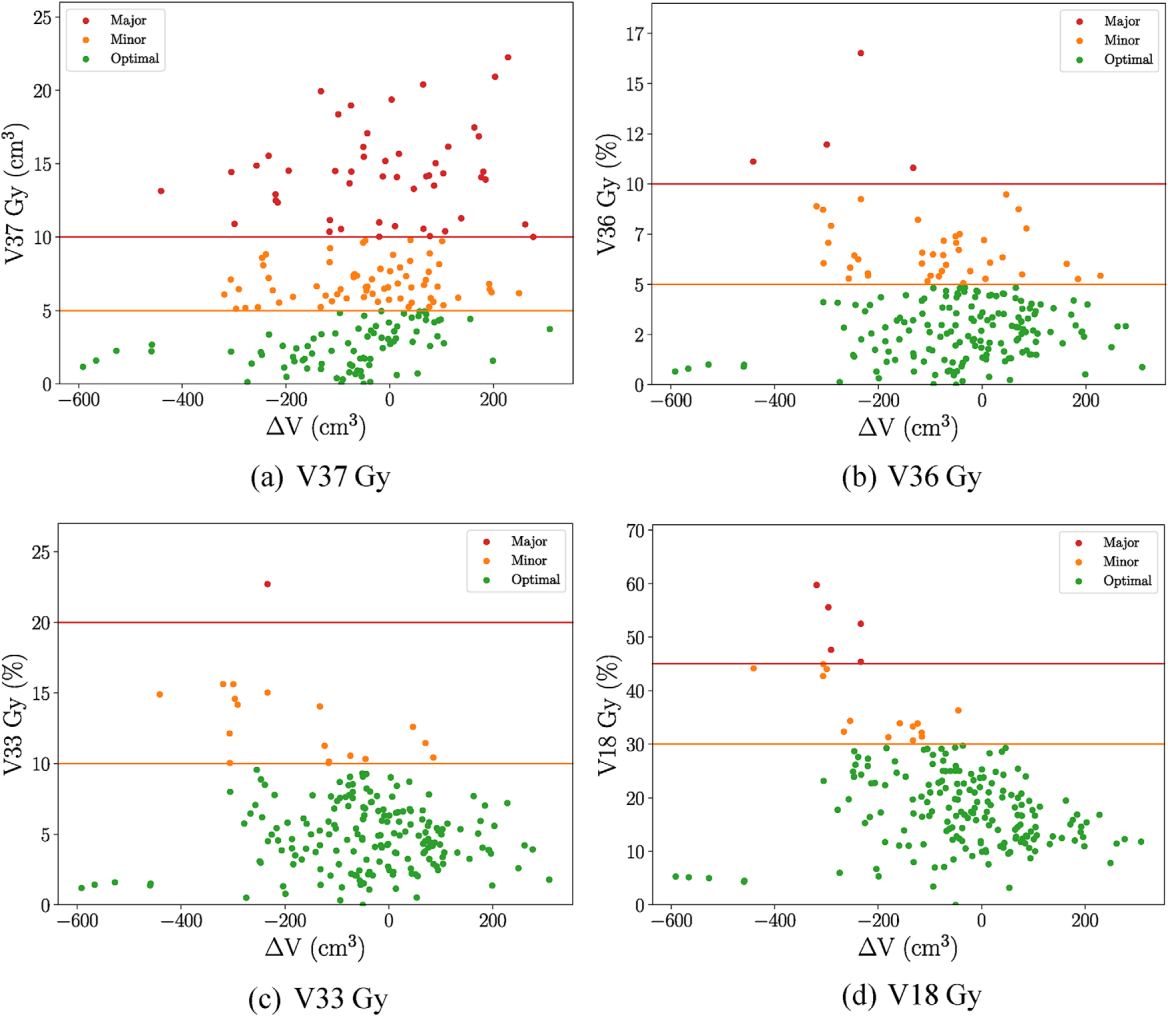
Bladder—Dose‐volume metrics compared to absolute change in volume from pCT to CBCT *(V*_CBCT_ *—V*_pCT_). CBCT, cone‐beam computed tomography; pCT, planning CT.

Figure 7’s scatter plots demonstrate the relationship between dose‐volume metrics and changes in bladder volume from CT to CBCT (*∆V*). Major violations in V18, V33, and V36 Gy metrics occurred when CBCT bladder volume was smaller than the pCT on account of the relative nature of these metrics. The V18, V33, and V36 Gy metrics are percent‐volume metrics and are directly influenced by changes in total volume. If the same absolute partial volume is irradiated to a certain dose, a decrease in total bladder volume would increase the percent value of the metric. In contrast, V37 Gy metric violations occurred across a range of *∆V* values. These were linked to bladder wall movement toward the PTV, particularly in the inferior and/or posterior directions. For V37 Gy, the direction of bladder displacement mattered more than the volume change from the pCT.

**FIGURE 8:**
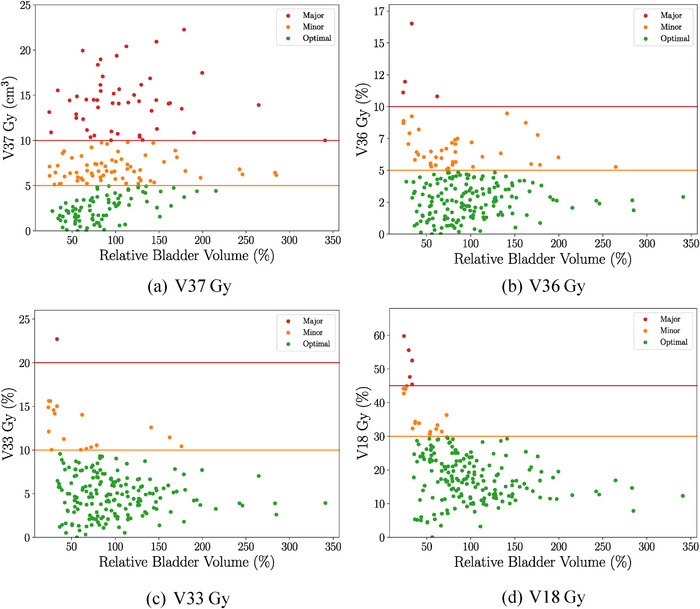
Bladder—Dose‐volume metrics compared to the relative change in volume from pCT to CBCT (*^V^*_CBCT_ */V*_pCT_ × 100%). CBCT, cone‐beam computed tomography; pCT, planning CT.

Additional findings indicate that in 59 out of the 200 fractions, the bladder volume at treatment was less than 2/3 of the pCT volume (Figure 8). The bladder decision tree (Figure 1) recommends the bladder volume at treatment be at least 2/3 of the pCT volume. However, only 23 out of these 59 instances were accurately identified by the radiation therapists at the time of treatment. All major violations for V18, V33, and V36 Gy metrics took place when the bladder volume was less than 2/3 of the pCT volume. Out of 50 fractions with major violations in the crucial V37 Gy metric, 10 had significantly reduced bladder volumes, but only four were correctly flagged as B1N (Bladder volume < ⅔ pCT volume, no increase in small bowel volume within 80% isodose) by therapists and treated without requiring RO follow‐up. The remaining six were misclassified as B2 (bladder volume > ⅔ pCT volume), thus incorrectly omitting the required bowel dose review prior to treatment. One patient, with a large pCT bladder volume of 577 cm^3^, had a V37 Gy major violation in every fraction. The data suggests that this patient struggled to replicate their pCT bladder volume during treatment, with CBCT volumes ranging from 66.2% to 23.6% of the pCT volume.

### 3.5 Rectum

**FIGURE 9:**
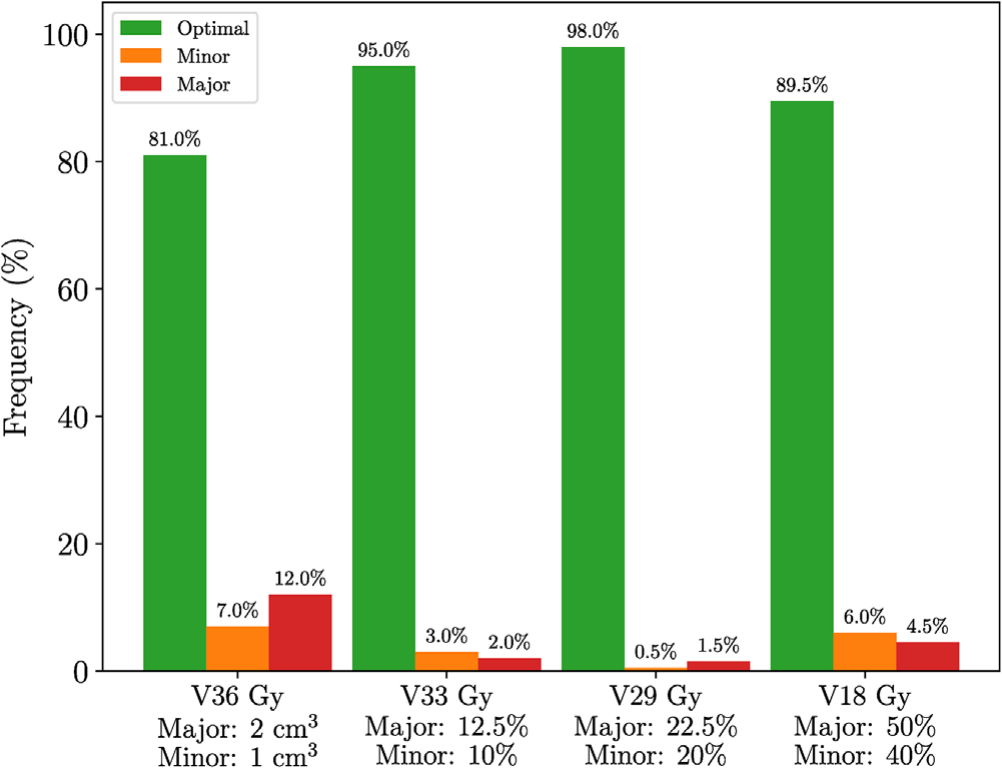
Rectum—Classification of dose‐volume metrics using daily CBCT rectum contours. CBCT, cone‐beam computed tomography.

In the initial treatment plans for all 40 patients, no rectum metrics showed minor violations. Yet, despite meticulous bowel and bladder preparation and high‐quality treatment plans, Figure 9 reveals minor and major violations during treatment delivery. Furthermore, a number of major and minor violations occurred for the “high‐dose” V36 Gy metric. Major violations for V18, V29, and V33 Gy were below 5%, while 12% had V36 Gy major violations. Minor violations for V29 and V33 Gy were low at 0.5% and 3.0% respectively; however the V18 and V36 Gy were at 6.0% and 7.0%. Overall, 14% of the 200 fractions had at least one major violation, 10% had at least one minor violation, and 76% were classified as optimal.

**FIGURE 10:**
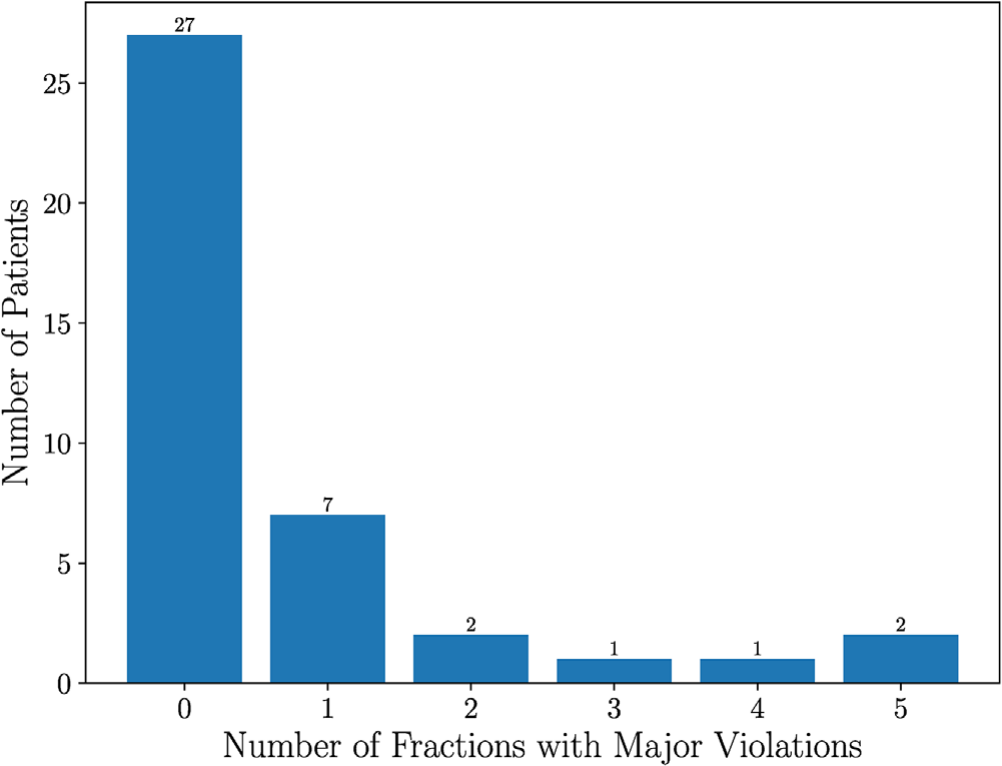
Rectum—Patients by number of major dose violations.

Despite most patients having optimal rectum metrics, some patients had systematic major dose‐volume violations (Figure 10). The two patients who had five fractions with major violations, and the one patient who had four, had their violations occur in the V36 Gy metric. These high dose‐volume metrics are associated with worse EPIC bowel QOL and grade 2 or higher Common Terminology Criteria for Adverse Events (CTCAE) hematochezia.^39^

**FIGURE 11:**
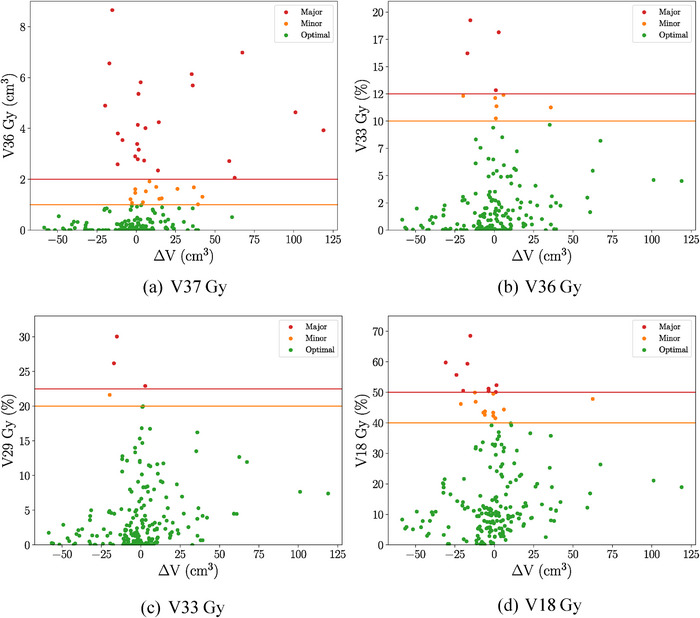
Rectum—Dose‐volume metrics compared to absolute change in volume from pCT to CBCT (*V*_CBCT_−*V*_pCT_). CBCT, cone‐beam computed tomography; pCT, planning CT.

Figure 11 shows that major violations for V18, V29, and V33 Gy mainly occurred at approximately *∆V*_rectum_ = 0 cm^3^, while larger volumes often resulted in optimal classifications. These larger volumes were more often classified as optimal; large OAR volumes in relative metrics would have a reduced sensitivity to dose because an increase in volume would decrease the metric's value for a fixed absolute irradiated partial volume. In contrast, the absolute V36 Gy metric showed major violations across a range of volumes.

Fractions with major violations tended to have anterior rectum distension as compared to the pCT, 70.8% of V36 Gy, all V33 Gy, all V29 Gy, and 55.6% of V18 Gy major violations occurred when the CBCT rectum contour distended anteriorly past the pCT rectum + 5 mm structure on at least three slices. This indicates that the rectum DT (Figure 2) when applied correctly, flags at least the majority of patients' setups that are problematic for rectal dose. However, in reality, only 25% of V36 Gy and V33 Gy, and 33.3% of V29 Gy and V18 Gy major violations were identified by therapists to have rectums that distended anteriorly past the pCT rectum + 5 mm structure and flagged for closer review.

## 4 DISCUSSION

In this study of 40 prostate SABR patients receiving 40 Gy in five fractions, Limbus‐generated CBCT contours were used to estimate per‐fraction DVH for the bladder and rectum. The results indicate that despite meticulous planning and adherence to preparation protocols, major violations were present in both bladder and rectum metrics, occurring in 27% and 14% of fractions, respectively. High‐dose, absolute volume metrics had the highest rates of major violations—25% for the bladder and 12% for the rectum. Of particular concern were four patients who had bladder V37 Gy major violations in all fractions, and two had rectum V36 Gy major violations in all fractions. These High‐dose, absolute volume metric violations were not strongly correlated with changes in OAR volume between pCT and CBCT but were more associated with organ deformations in proximity to high‐dose regions on CBCT. When a major violation occurred, comparing CBCT and pCT rectum contours showed anterior rectum distension at treatment in 70.8% of V36 Gy violations, all V33 Gy and V29 Gy violations, and 50.6% of V18 Gy violations.

Analyzing the adherence to the clinical DT also showed that therapists struggled to follow the correct DT pathway. In 59 out of 200 fractions where bladder volume at treatment was less than two‐thirds of the volume observed in the pCT, only 23 instances were correctly identified as category B1Y/N by the treating therapists meaning small bowel dose was not evaluated in 36 fractions as required by protocol. For the rectum, our analysis showed that the correct application of the DT should have flagged a majority of the fractions that had major rectal dose violations but in reality, only 25% of V36 Gy and V33 Gy, and 33.3% of V29 Gy and V18 Gy major violations were labeled by therapists as SR2 or SR3 and tagged for closer review. Reasons for the discrepancies in adhering to the DTs may include difficulty in visually estimating two‐thirds of a bladder's volume from two‐dimensional image slices (sagittal, coronal, and axial planes), time pressure to quickly scroll through image slices and evaluate rectal distension, and reluctance to trigger action thresholds that could extend the patient time slot in a busy clinic. All these results point to a strong need for an online automated tool to provide quantitative dose metrics in the evaluation of patient setup because not only are the current clinical DTs difficult for staff to follow but the simple visual thresholding rules are not sensitive enough to alert clinicians to important dose constraint violations for every patient.

The dose analysis of this study was similar to one conducted by Devlin et al.,^2^ which also used the approach of overlaying a planned dose onto CBCT contours for dose‐volume metric evaluation during treatment. Devlin et al., focused on prostate SABR patients treated with 35 Gy in five fractions using VMAT without SpaceOAR gel; contouring for their study was also performed manually. For bladder, the group saw a negative correlation between their high dose V35 Gy < 1% dose metric and bladder volume. For the rectum, they observed a positive correlation between rectum volume and their high dose V35 Gy < 1% metric. In contrast, this study used absolute volume for the high dose metrics for bladder (V37 Gy ≤ 5 cm^3^) and rectum (V36 Gy ≤ 1 cm^3^) and showed no clear volume relationship (Figure 8), despite the addition of SpaceOAR gel. Only organ deformations close to the target were indicative of increased OAR dose.

The OAR volume change analysis of this study was generally in line with other published studies confirming that significant OAR deformation is common despite attempts to minimize it through various patient bladder and bowel preparation protocols. Fuchs et al.^40^ studied dose to OARs in prostate treatments using a Tomotherapy unit. Their CBCT bladder and rectum volumes respectively ranged from 22% to 375% and 62% to 223% of the initial plan volume, while this study's bladder and rectum volumes respectively ranged from 40.1% to 185.7% and 57.0% to 230.8%. Tanny et al.,^41^ studying OAR doses using a CBCT‐guided online adaptive RT platform, saw significant mean deviations ([*V*_CBCT_ *−V*_pCT_]/*V*_pCT_ × 100%) in the bladder and rectum. In their study, bladder and rectum deviations ranged between −47% to +12% and −31% to +16% respectively, whereas this study saw deviations range from −59.9% to 85.7% (average −3.5%, standard deviation of 40%) and −42.9%–130.8% (average +5.1%, standard deviation of 31.2%). The greater deviation range in this study could be due to patients receiving five fractions compared to the 25 fractions in the study by Tanny et al. Patients receiving 25 fractions of RT would have more practice with their bowel/bladder preparation protocol, reducing the overall variability of OAR volumes.

This study had several limitations. First, the planned dose was superimposed on the CBCT rather than performing a full dose recalculation which introduces errors in our dose estimates. For prostate treatment using multiple beams and where the tissue near the target is fairly homogeneous, the dose errors are within clinically acceptable standards.^32^ However, the validity of the dose cloud shift and deformation‐invariance for a small proportion of patients with hip prostheses may not hold and should be investigated in a future study. A second limitation of this study was that each fraction is treated as if it delivers the full prescription dose. This was done to facilitate the classification of metrics into optimal, minor, or major categories according to planning goals. Dose accumulation, where individual voxels are tracked across the course of treatment using DIR, was not performed. Accurate DIR requires thorough testing and evaluation with deformable phantom measurements before it can be clinically implemented.^42^, ^43^ Due to the variety of vendor DIR algorithms and lack of patient ground truth, many clinicians are not comfortable using dose accumulation and still prefer to evaluate dose distributions per fraction.

Finally, the third limitation is that at the time of this study, Limbus did not support small bowel and target auto‐contouring. Small bowel could be difficult to auto‐contour for this study's dataset as the narrow field of view used for the CBCTs often excluded parts of the bowel, especially for patients with large bladder volumes. CTV/PTV auto‐contouring would also be beneficial and can be incorporated in future studies. However, with the standard use of implanted fiducials for prostate SABR patients, our clinicians are comfortable with tumor targeting and are more concerned with OAR dose sparing.

Despite these limitations, our method is fast and accurate enough for most patients to constitute a clinically feasible tool for patient setup decision‐making at the treatment console. It is intended to be used in clinics that do not currently have plans to implement cutting‐edge online adaptive units such as the Elekta Unity MR‐Linac (Elekta AB, Stockholm, Sweden) or the Varian Ethos. While online adaptive radiotherapy systems offer more precise dose adjustments, implementation of this technology presents significant challenges in terms of resource allocation, including staff training, staffing levels, and equipment costs.^44^ Globally, several studies highlight a critical shortage of both staff and equipment to meet current cancer radiotherapy demands, not including the increased resources required for online adaptive therapy.^45^, ^46^, ^47^ Assuming global radiotherapy needs are met, even a simple shift from conventional fractionation toward hypofractionation could potentially result in operational cost savings of $2.55 billion for prostate cancer per year.^47^ Transitioning to prostate SABR fractionation for the appropriate subset of patients would lead to even greater efficiencies. However, the implementation of SABR treatments with its higher dose per fraction also entails its own set of stricter requirements across the entire patient workflow from machine QA to treatment planning and delivery. Safe and effective guidance for prostate SABR patient setup is one key component in this chain. Given the resource constraints that will likely prevent many institutions from adopting online adaptive therapy equipment in the near future, our online automatic tool can bridge this gap by offering quick feedback to therapists and reducing reliance on qualitative assessments or delayed post‐treatment reviews.

This study laid the groundwork for an online decision support tool for SABR prostate patient setup at a center that does not have online adaptive capabilities. The next step is to integrate this software program into new bladder and rectum DTs where patient setup decisions are based on anticipated delivered doses rather than solely relying on visual CBCT assessments. Patients typically waited between 2–6 min on the treatment couch for therapists to determine if the patient was adequately prepared by navigating the bladder and rectum DTs. During this time, bladder filling can change significantly.^48^, ^49^ The software tool has the potential to reduce the time between the pre‐treatment CBCT and the first arc to less than a minute by automating patient setup assessment, further reducing intrafraction bladder filling uncertainties. An online solution involving auto‐generated contours would require QA processes incorporated into the workflow which would be investigated in future work. With further optimization of the code's computational time, the more ambitious aim is to evolve toward an adaptive positioning RT solution. Rather than a full re‐plan for each fraction, the program could automatically calculate small 3D/6D couch shifts to enhance patient setup, thereby minimizing the likelihood of dose violations. Given the observed major violations in bladder V37 Gy and rectum V36 Gy metrics—often occurring when the OAR was distended toward the target volume relative to the pCT—the capacity for couch shifts based on estimated dose could likely mitigate OAR exposure. Although this may not achieve the dosimetric efficiency of a fully adaptive RT system, it represents a feasible short‐term goal that does not necessitate extended patient treatment time or additional financial resources.

## 5 CONCLUSION

This study lays the foundation for a rapid, decision‐support tool to assist therapists in making informed patient setup decisions in prostate SABR. By providing quantitative dose metrics, the tool enhances the consistency of setup evaluations and reduces the risk of major dose‐volume violations. Unlike adaptive radiotherapy systems, which require significant resource investments, this tool integrates seamlessly into standard clinical workflows. Our findings indicate that Bladder and rectum dose‐volume metrics at treatment were higher than were anticipated during treatment planning. Variability in OAR volume and position during treatment, even with stringent preparation protocols, appeared to contribute to these metric violations. Current population‐based, qualitative DTs may be insufficient for prostate SABR cases due to the high per‐fraction dose and associated toxicity risk. DTs lack sensitivity to all but the most extreme anatomical variations, leading to unrecognized violations of dose constraints. Furthermore, the presence of human error in applying DTs results in inconsistent decision‐making. For example, only 23 of 59 underfilled bladders were correctly flagged for review, demonstrating the limitations of qualitative assessment. By introducing a quantitative, automated tool, we provide a more consistent and objective approach to assessing patient setup, allowing for quantitative dose metrics to better inform clinicians and replace the current visual estimation used in online decision‐making.

## AUTHOR CONTRIBUTIONS

Erika Chin conceptualized and supervised the project. Conor Sinclair Smith developed the scripts for CBCT OAR DVH calculations, conducted the dose shift study, and was the primary author of the manuscript. Karl Otto, Carter Kolbeck, and Joshua Giambattista developed the CBCT autocontouring software. Abraham Alexander, Sonja Murchison, and Andrew Pritchard provided clinical expertise and contouring corrections. All co‐authors revised and approved the final manuscript.

## CONFLICT OF INTEREST STATEMENT

The authors declare no conflicts of interest.

## References

[1] Choudhury A , Henry MFA , Mitin MPT , Chen MMR , Joseph MFN , Spratt MPDE . Photons, protons, sbrt, brachytherapy—what is leading the charge for the management of prostate cancer? a perspective from the gu editorial team. Int J Radiat Oncol Biol Phys. 2021;110(4):1114‐1121. doi:10.1016/j.ijrobp.2021.01.003 34171236

[2] Devlin L , Dodds D , Sadozye A , et al. Dosimetric impact of organ at risk daily variation during prostate stereotactic ablative radiotherapy. Br J Radiol. 2020;93(1108). doi:10.1259/bjr.20190789 PMC7362910 31971829

[3] Alexander A , Kwan W . Androgen Suppression With Stereotactic body or external beam radiation therapy (ASSERT): a phase II randomized trial for intermediate and high risk prostate cancer. ClinicalTrials.gov identifier: NCT02594072. Accessed May 31, 2024. https://clinicaltrials.gov/study/NCT02594072?tab=table

[4] Gagne I , Earnshaw K , Cowan S , Goulart J , Alexander A . OC‐0530 improving OAR volumes during prostate RT using daily patient feedback and standardized protocols. Radiot Oncol. 2019;133:S279. doi:10.1016/S0167-8140(19)30950-8

[5] Imae T , Haga A , Watanabe Y , et al. Retrospective dose reconstruction of prostate stereotactic body radiotherapy using cone‐beam CT and a log file during VMAT delivery with flattening‐filter‐free mode. Radiol Phys Technol. 2020;13(3):238‐248. doi:10.1007/s12194-020-00574-3 32656744

[6] Orlandini LC , Coppola M , Fulcheri C , Cernusco L , Wang P , Cionini L . Dose tracking assessment for image‐guided radiotherapy of the prostate bed and the impact on clinical workflow. Radiat Oncol. 2017;12(1):78. doi:10.1186/s13014-017-0815-y 28454559 PMC5410096

[7] Greep JM , Siezenis LMLC . Methods of decision analysis: protocols, decision trees, and algorithms in medicine. World J Surg. 1989;13(3):240‐244. doi:10.1007/BF01659028 2662622

[8] Osong B , Bermejo I , Lee KC , Lee SH , Dekker A , van Soest J . Prediction of radiotherapy compliance in elderly cancer patients using an internally validated decision tree. Cancers. 2022;14(24):6116. doi:10.3390/cancers14246116 36551602 PMC9776371

[9] Putora PM , Panje CM , Papachristofilou A , Pra AD , Hundsberger T , Plasswilm L . Objective consensus from decision trees. Radiat Oncol. 2014;9(1):270. doi:10.1186/s13014-014-0270-y 25476988 PMC4269842

[10] Ng CKC , Leung VWS , Hung RHM . Clinical evaluation of deep learning and atlas‐based auto‐contouring for head and neck radiation therapy. Appl Sci. 2022;12(22):11681. doi:10.3390/app122211681

[11] Ahn SH , Yeo AU , Kim KH , et al. Comparative clinical evaluation of atlas and deep‐learning‐based auto‐segmentation of organ structures in liver cancer. Radiat Oncol. 2019;14(1):213. doi:10.1186/s13014-019-1392-z 31775825 PMC6880380

[12] Zabel WJ , Conway JL , Gladwish A , et al. Clinical evaluation of deep learning and atlas‐based auto‐contouring of bladder and rectum for prostate radiation therapy. Pract Radiat Oncol. 2021;11(1):e80‐e89. doi:10.1016/j.prro.2020.05.013 32599279

[13] Chen W , Wang C , Zhan W , et al. A comparative study of auto‐contouring softwares in delineation of organs at risk in lung cancer and rectal cancer. Sci Rep. 2021;11(1):23002. doi:10.1038/s41598-021-02330-y 34836989 PMC8626498

[14] Starke A , Poxon J , Patel K , et al. Clinical evaluation of the efficacy of limbus artificial intelligence software to augment contouring for prostate and nodes radiotherapy. British J Radiol. 2024;97(1158):1125‐1131. doi:10.1093/bjr/tqae077 PMC11135797 38627245

[15] Marin Anaya V . Artificial intelligence based auto‐contouring solutions for use in radiotherapy treatment planning of head and neck cancer. IPEM‐Translation. 2023;6‐8:100018. doi:10.1016/j.ipemt.2023.100018

[16] Barta R , Nygren I , Kirkby C , Shaw C , See S . Qualitative evaluation of AI contours. Poster presented at: CARO‐COMP Joint Scientific Meeting; September 2023; Montreal, QC; Virtual; Accessed February 2. 2025. https://www.cureus.com/posters/2554‐qualitative‐evaluation‐of‐ai‐contours

[17] Radici L , Ferrario S , Borca VC , et al. Implementation of a commercial deep learning‐based auto segmentation software in radiotherapy: evaluation of effectiveness and impact on workflow. Life. 2022;12(12):2088. doi:10.3390/life12122088 36556455 PMC9782080

[18] Kim YW , Biggs S , Claridge Mackonis E . Investigation on performance of multiple AI‐based auto‐contouring systems in organs at risks (OARs) delineation. Phys Eng Sci Med. 2024;47(3):1123‐1140. doi:10.1007/s13246-024-01434-9 39222214 PMC11408550

[19] D'Aviero A , Re A , Catucci F , et al. Clinical validation of a deep‐learning segmentation software in head and neck: an early analysis in a developing radiation oncology center. Int J Environ Res Public Health. 2022;19(15):9057. doi:10.3390/ijerph19159057 35897425 PMC9329735

[20] Wong J , Huang V , Giambattista JA , et al. Training and validation of deep learning‐based auto‐segmentation models for lung stereotactic ablative radiotherapy using retrospective radiotherapy planning contours. Front Oncol. 2021;11:626499. doi:10.3389/fonc.2021.626499 34164335 PMC8215371

[21] O'Neill AGM , Jain S , Hounsell AR , O'Sullivan JM . Fiducial marker guided prostate radiotherapy: a review. Br J Radiol. 2016;89(1068):20160296. doi:10.1259/bjr.20160296 27585736 PMC5604907

[22] Skarsgard D , Cadman P , El‐Gayed A , et al. Planning target volume margins for prostate radiotherapy using daily electronic portal imaging and implanted fiducial markers. Radiat Oncol. 2010;5(1):52. doi:10.1186/1748-717X-5-52 20537161 PMC2896366

[23] Zelefsky MJ , Kollmeier M , Cox B , et al. Improved clinical outcomes with high‐dose image guided radiotherapy compared with non‐igrt for the treatment of clinically localized prostate cancer. Int J Radiat Oncol Biol Phys. 2012;84(1):125‐129. doi:10.1016/j.ijrobp.2011.11.047 22330997

[24] Radici L , Piva C , Casanova Borca V , et al. Clinical evaluation of a deep learning CBCT auto‐segmentation software for prostate adaptive radiation therapy. Clin Transl Radiat Oncol. 2024;47:100796. doi:10.1016/j.ctro.2024.100796 38884004 PMC11176659

[25] Chetty IJ , Cai B , Chuong MD , et al. Quality and safety considerations for adaptive radiation therapy: an ASTRO white paper. Int J Radiat Oncol Biol Phys. 2024;0(0). doi:10.1016/j.ijrobp.2024.10.011. Published online October 2024. 39424080

[26] Smyth G , McCallum HM , Lambert EL , Lawrence GP . A dose distribution overlay technique for image guidance during prostate radiotherapy. Br J Radiol. 2008;81(971):890‐896. doi:10.1259/bjr/55773072 18824502

[27] Smyth G , McCallum HM , Pearson MJM , Lawrence GP . Comparison of a simple dose‐guided intervention technique for prostate radiotherapy with existing anatomical image guidance methods. Br J Radiol. 2012;85(1010):127‐134. doi:10.1259/bjr/13032912 21385920 PMC3473952

[28] Pukala J , Staton R , Langen K , SU‐E‐J‐201 . What is the importance of dose recalculation for adaptive radiotherapy dose assessment?. Med Phys. 2012;39(6Part9):3699. doi:10.1118/1.4735041 28519011

[29] Pukala J , Gray T , Meeks SL , Manon RR , Staton RJ . Adaptive radiation therapy replanning for head‐and‐neck cancers and the dosimetric benefit to the parotid glands. Int J Radiat Oncol Biol Phys. 2013;87(2):S713‐S714. doi:10.1016/j.ijrobp.2013.06.1890

[30] Qi XS , Santhanam A , Neylon J , et al. Near real‐time assessment of anatomic and dosimetric variations for head and neck radiation therapy via graphics processing unit‐based dose deformation framework. Int J Radiat Oncol Biol Phys. 2015;92(2):415‐422. doi:10.1016/j.ijrobp.2015.01.033 25847607

[31] Papalazarou C , Klop GJ , Milder MTW , et al. CyberKnife with integrated CT‐on‐rails: system description and first clinical application for pancreas SBRT. Med Phys. 2017;44(9):4816‐4827. doi:10.1002/mp.12432 28657157

[32] Sharma M , Weiss E , Siebers JV . Dose deformation‐invariance in adaptive prostate radiation therapy: implication for treatment simulations. Radiother Oncol. 2012;105(2):207‐213. doi:10.1016/j.radonc.2012.10.011 23200409 PMC6559364

[33] Brock KK , Mutic S , McNutt TR , Li H , Kessler ML . Use of image registration and fusion algorithms and techniques in radiotherapy: report of the AAPM radiation therapy committee task group no. 132. Med Phys. 2017;44(7):e43‐e76. doi:10.1002/mp.12256 28376237

[34] Maraghechi B , Mazur T , Lam D , et al. Phantom‐based quality assurance of a clinical dose accumulation technique used in an online adaptive radiation therapy platform. Adv Radiat Oncol. 2023;8(3):101138. doi:10.1016/j.adro.2022.101138 36691450 PMC9860416

[35] Zhong H , Pursley JM , Rong Y . Deformable dose accumulation is required for adaptive radiotherapy practice. J Appl Clin Med Phys. 2024;25(8):e14457. doi:10.1002/acm2.14457 39031438 PMC11302806

[36] Biggs S , Jennings M , Swerdloff S , et al. PyMedPhys: a community effort to develop an open, Python‐based standard library for medical physics applications. J Open Source Softw. 2022;7(78):4555. doi:10.21105/joss.04555

[37] Miften M , Olch A , Mihailidis D , et al. Tolerance limits and methodologies for IMRT measurement‐based verification QA: recommendations of AAPM Task Group No. 218. Med Phys. 2018;45(4):e53‐e83. doi:10.1002/mp.12810 29443390

[38] Hadigal SR , Gupta AK . Application of hydrogel spacer SpaceOAR Vue for prostate radiotherapy. Tomography. 2022;8(6):2648‐2661. doi:10.3390/tomography8060221 36412680 PMC9680261

[39] Wang K , Mavroidis P , Royce TJ , et al. Prostate stereotactic body radiation therapy: an overview of toxicity and dose response. Int J Radiat Oncol Biol Phys. 2021;110(1):237‐248. doi:10.1016/j.ijrobp.2020.09.054 33358229 PMC8053668

[40] Fuchs F , Habl G , Devečka M , Kampfer S , Combs SE , Kessel KA . Interfraction variation and dosimetric changes during image‐guided radiation therapy in prostate cancer patients. Radiat Oncol J. 2019;37(2):127‐133. doi:10.3857/roj.2018.00514 31137087 PMC6610012

[41] Tanny S , Sperling NN , Zheng D , et al. Tracking OAR volume and DVH variability in the initial cohort of pelvic patients treated with CBCT‐guided, online adaptive therapy. Int J Radiat Oncol Biol Phys. 2022;114(3):e598. doi:10.1016/j.ijrobp.2022.07.2290

[42] Xiao H , Ren G , Cai J . A review on 3D deformable image registration and its application in dose warping. Radiat Med Prot. 2020;1(4):171‐178. doi:10.1016/j.radmp.2020.11.002

[43] Oh S , Kim S . Deformable image registration in radiation therapy. Radiat Oncol J. 2017;35(2):101‐111. doi:10.3857/roj.2017.00325 28712282 PMC5518453

[44] McComas KN , Yock A , Darrow K , Shinohara ET . Online adaptive radiation therapy and opportunity cost. Adv Radiat Oncol. 2023;8(3):101034. doi:10.1016/j.adro.2022.101034 37273924 PMC10238262

[45] Zhu H , Chua MLK , Chitapanarux I , et al. Global radiotherapy demands and corresponding radiotherapy‐professional workforce requirements in 2022 and predicted to 2050: a population‐based study. Lancet Glob Health. 2024;12(12):e1945. doi:10.1016/S2214-109X(24)00355-3 39401508

[46] Atun R , Jaffray DA , Barton MB , et al. Expanding global access to radiotherapy. Lancet Oncol. 2015;16(10):1153‐1186. doi:10.1016/S1470-2045(15)00222-3 26419354

[47] Abdel‐Wahab M , Giammarile F , Carrara M , et al. Radiotherapy and theranostics: a Lancet Oncology Commission. Lancet Oncol. 2024;25(11):e545‐e580. doi:10.1016/S1470-2045(24)00407-8 39362232

[48] Krishnan A , Tsang YM , Stewart‐Lord A . The impact of intra‐fractional bladder filling on “Plan of the day” adaptive bladder radiotherapy. Tech Innov Patient Support Radiat Oncol. 2019;9:31‐34. doi:10.1016/j.tipsro.2019.01.001 32095593 PMC7033786

[49] Foroudi F , Pham D , Bressel M , Gill S , Kron T . Intrafraction bladder motion in radiation therapy estimated from pretreatment and posttreatment volumetric imaging. Int J Radiat Oncol Biol Phys. 2013;86(1):77‐82. doi:10.1016/j.ijrobp.2012.11.035 23332382

